# Effect of nano-curcumin supplementation on angina status, and traditional and novel cardiovascular risk factors in overweight or obese patients with coronary slow flow phenomenon: a randomized double-blind placebo-controlled clinical trial

**DOI:** 10.1186/s40795-024-00877-3

**Published:** 2024-05-13

**Authors:** Mahsa Rezaei, Mitra Soltani, Elham Alipoor, Seyed Mahdi Rezayat, Ali Vasheghani-Farahani, Mehdi Yaseri, Ata Firouzi, Mohammad Javad Hosseinzadeh-Attar

**Affiliations:** 1 Department of Clinical Nutrition, School of Nutritional Sciences and Dietetics, Tehran University of Medical Sciences, Tehran, Iran; 2 Department of Pharmacology, School of Medicine, Tehran University of Medical Sciences, Tehran, Iran; 3 Department of Nanomedicine, School of Advanced Technologies in Medicine, Tehran University of Medical Sciences, Tehran, Iran; 4 Cardiac Primary Prevention Research Center, Cardiovascular Diseases Research Institute, Tehran University of Medical Sciences, Tehran, Iran; 5 Department of Clinical Cardiac Electrophysiology, Tehran Heart Center, Tehran University of Medical Sciences, Tehran, Iran; 6 Department of Epidemiology and Biostatistics, School of Public Health, Tehran University of Medical Sciences, Tehran, Iran; 7 Rajaie Cardiovascular, Medical & Research Center, Iran University of Medical Sciences, Tehran, Iran

**Keywords:** Coronary slow flow phenomenon, Curcumin, Angina status, Cardiovascular risk factors, Endocan, Adropin, Homocysteine

## Abstract

**Background:**

Cardiovascular events and poor quality of life are frequently observed in patients with coronary slow flow phenomenon (CSFP). This trial evaluated the effect of nano-curcumin supplement containing curcuminoids, as multifunctional nutraceuticals, on angina status, and some traditional and novel cardiovascular risk factors in overweight or obese patients with CSFP.

**Methods:**

In this double-blind, randomized, placebo-controlled clinical trial, 42 overweight or obese patients with CSFP received either 80 mg/day of nano-curcumin or placebo for 12 weeks. Seattle angina questionnaire (SAQ) as a clinical measure of angina status, circulating endocan, adropin, homocysteine, lipid profile, and the novel scores of visceral adiposity index (VAI) and waist-triglyceride index (WTI) were assessed before and after the intervention. The independent samples t-test, Mann-Whitney test, analysis of covariance, Chi-square, and Fisher’s exact tests were used where appropriate.

**Results:**

All domains of SAQ including physical limitation, angina stability, angina frequency-severity, treatment satisfaction, and disease perception and quality of life improved significantly in the nano-curcumin compared with the placebo group. No significant changes were observed in serum endocan, adropin, and homocysteine following the intervention. Triglycerides, triglyceride/high-density lipoprotein cholesterol ratio, WTI and VAI values improved significantly only within the nano-curcumin group.

**Conclusions:**

Supplementation with 80 mg/day nano-curcumin (containing curcuminoids) for 12 weeks significantly improved clinically important disease-specific aspects of health in patients with CSFP. Some traditional and novel cardiovascular risk factors improved significantly only compared with the baseline values, which need further investigation.

**Trial registration:**

This study was approved by the Ethics Committee of Tehran University of Medical Sciences (IR.TUMS.VCR.REC.1398.794). The study protocol was registered at Iranian Registry of Clinical Trials by IRCT20131125015536N8 registration ID at 19.06.2019.

**Supplementary Information:**

The online version contains supplementary material available at 10.1186/s40795-024-00877-3.

## Introduction

Coronary slow flow phenomenon (CSFP) is a clinical condition documented by angiography as delayed distal opacification of coronary vessels without any significant stenosis [1]. Patients with CSFP would experience recurrent chest pain, even at rest, repeated hospitalizations, and readmissions to cardiac units [2]. Life-threatening arrhythmia and sudden cardiac death have been reported as well [3]. Despite the largely unknown pathogenesis, some potential mechanisms including microvascular disease, inflammation, and endothelial dysfunction have been suggested to be involved in CSFP [2, 4].

Endothelial dysfunction is a hallmark of many cardiovascular diseases including CSFP [2, 5]. The imbalance between vasoconstrictors and vasodilators such as reduced nitric oxide (NO) bioavailability and increased endothelin-1, as well as decreased circulating adropin and elevated homocysteine and endocan have been indicated in this disorder [2, 6–8]. These changes in homocysteine, endocan, and adropin concentrations were reported as independent predictors of the presence and severity of CSFP [6–8].

Increased homocysteine levels, a well-known cardiovascular risk factor, can induce inflammation and endothelial dysfunction, and are correlated with reduced adropin levels [9]. Adropin and endocan have been suggested as novel regulators of endothelial function [10, 11]. Adropin is a peptide hormone that exerts a potential protective effect on endothelium mainly through upregulation of the enzyme nitric oxide synthase (NOS) and hence NO bioavailability [11, 12]. Endocan, also known as endothelial cell-specific molecule-1, is expressed in several tissues including endothelial cells, which regulates cell adhesion [13]. Endocan expression in activated endothelial cells, as well as its serum concentrations, have been elevated in some inflammatory diseases proportional to the disease severity, which makes it a potential predictor of clinical outcomes [13–15].

Currently, few clinical studies are available investigating effective treatments in CSFP due to poorly known pathogenesis and relatively low prevalence of the condition, which complicate conducting well-designed clinical trials. However, CSFP has a clinical significance and can considerably disturb the quality of life of the patients [16].

Nutraceutical compounds especially plant-based substances are among the most studied interventions in different cardiovascular diseases, as they have the potential to affect many cardiometabolic risk factors [17]. Turmeric has been widely used as a food additive and also as a medicinal plant [18]. Turmeric has three active chemical components called curcuminoids (approximately 77% curcumin, 17% demethoxycurcumin, and 3% bisdemethoxycurcumin) [19]. Most of the pharmacological activities of turmeric have been attributed mainly to curcuminoids. Curcuminoids have shown antioxidant, anti-cancer, anti-inflammatory and cardioprotective activities [20]. Curcumin is an active ingredient and the major compound of the plant Curcuma longa (turmeric) [18]. Nano-curcumin is a nano-formulation of curcumin to address the concerns about poor bioavailability and increase the potential efficacy of this compound [21]. Several studies that investigated the effect of curcumin on endothelial dysfunction have shown promising results [22, 23]. Curcumin may increase NOS activity and NO bioavailability, decrease the expression of endothelial leukocyte adhesion molecules and prevent platelet adhesion to endothelial cells through anti-inflammatory activity, and improve flow-mediated dilation (FMD), a clinical measure of endothelial dysfunction [22, 24]. Additionally, curcumin supplementation has improved key cardiovascular risk factors including obesity and anthropometries, high blood pressure, and dyslipidemia in different medical conditions [25–27], which are suggested as independent predictors of CSFP [28, 29]. Moreover, curcumin has considerably improved bodily pain and quality of life in patients with liver cirrhosis and irritable bowel syndrome [30, 31]. However, to our knowledge, no previous study is available investigating the effect of curcuminoids supplementation on risk factors and clinical outcomes in CSFP. Thus, considering the proposed beneficial effects of curcuminoids on endothelial dysfunction and cardiovascular risk factors and the lack of studies on CSFP, this clinical trial was conducted to investigate the effect of a nano-curcumin supplement on angina status, and some of the conventional and novel cardiovascular risk factors in patients with CSFP.

## Methods

### Patients

The medical records of the patients coded as having CSFP in a referral heart hospital were rechecked by an interventional cardiologist based on the corrected thrombolysis in the myocardial infarction frame count method (CTFC), previously described by Gibson et al. [32]. Individuals with CTFC higher than 27 for any of the three main coronary arteries (right coronary artery, left circumflex artery, and left anterior descending artery) with less than 40% stenosis were invited to the study. Other eligibility criteria were overweight or obese adults (body mass index (BMI) ranged from ≥ 25 to < 40 kg/m^2^) aged 35–70 years, and left ventricular ejection fraction ≥ 45%. Individuals with the following criteria were not included in the study: those with any history of thyroid disorders, malignancies, pulmonary diseases, systemic and autoimmune diseases, renal or liver failure, cardiovascular diseases or anomalies, CSF secondary to revascularization procedure or interventions, active gastrointestinal bleeding and peptic ulcers, drug abuse or alcohol consumption as well as professional athletes or regular exercisers, premature menopause, and routine consumption of non-steroidal anti-inflammatory drugs, aphrodisiac medications, corticosteroids or immunosuppressants, anticonvulsants, multi-vitamins containing vitamin B_6_, B_9_, B_12_, and polyphenols, and omega 3 fatty acids (≥ 1 g/day).

### Study design

A parallel-design, randomized, double-blind, placebo-controlled clinical trial was used for this investigation. To place participants in the nano-curcumin and placebo groups, a stratified permuted block randomization scheme with block sizes of 2 and 4 was employed. A statistician unrelated to the sampling process supplied the randomization tables. Patients were categorized based on two variables: (1) gender and (2) cardiovascular risk level, due to the study’s multiple potential confounding factors. The risk level was calculated by denoting a point value of 1 for a family history of smoking, hypertension, diabetes, dyslipidemia, and coronary artery disease, and 0 for its absence. Additionally, ACE-I, ARBs, aspirin, beta-blockers, statins, anticoagulants, calcium channel blockers, nitrates, and aspirin were the medications that were assigned a point value of 0 if they were used, and 1 if they were not. Each patient’s points were added together, and the final score ran from 0 to 6. High risk was associated with scores greater than three, while scores up to three were regarded as low risk.

The intervention group received an 80 mg/day nano-curcumin capsule *(SinaCurcumin: Exir Nano Sina Co. Tehran, Iran)* and the placebo group received one placebo capsule daily for 12 weeks. According to several clinical trials, the previously tested and regarded as safe dose of nano-curcumin with no reported serious adverse reactions for adults in different medical conditions was 80 mg daily [33–36]. In addition, the nano-curcumin supplement contained curcuminoids. To formulate curcuminoid nanomicelles, curcuminoid, polysorbate surfactant, ascorbic acid, vitamin E, natural oils, and distilled water were used. Curcuminoids (curcumin, demethoxycurcumin, and bisdemethoxycurcumin) were determined according to the USP35. The mean diameter of nanomicelles was 9.5 ± 0.1 nm and AUC of nanomicellar curcuminoids was 59.2 times more than free curcuminoids [37]. Detailed information on curcumin nanomicelles is available at: *“*https://patentscope.wipo.int/*”* by the patent number “*PCT/IB2018/051370*” [38].

Placebo and nano-curcumin capsules were identical in appearance, size, smell, color, and packaging. The boxes were labeled as A or B. Both patients and main investigators were blinded to the assignment of the patients to the groups. The compliance rate was assessed by counting the returned capsules in weeks 6 and 12 (final) visits. Tolerability and adverse events were monitored by weekly phone calls. Patients not taking the supplements due to adverse reactions, not consuming more than 10% of the capsules, not attending follow-up sessions, or those reluctant to continue the study for any reasons were excluded from the trial.

The participants were required to take one capsule per day after one of the meals with reasonable time intervals away from other medicines. The patients were instructed to maintain their regular level of physical activity and diet throughout the study. All patients received standard medical treatments during the study period.

The research outline, goals, potential risks, and benefits were clarified and written informed consents were obtained from all participants before the study.

### Measurements

All measurements were performed at baseline and following 12 weeks of the intervention. Anthropometric indexes including weight and height were assessed with light clothing and barefoot to the nearest 0.1 kg and 0.5 cm, respectively, using a scale with a stadiometer (SECA, Germany). BMI was calculated by dividing weight (kg) by squared height (m^2^). Waist circumference (WC) was measured at the mid-point between the lowest rib and the top of the iliac crest to the nearest 0.5 cm using a flexible tape. Body composition was analyzed after overnight fasting with light clothing and an empty bladder using a bioelectrical impedance analysis device (InBody 770, USA), and blood pressure was measured using a digital device (B.Well, Switzerland) after adequate rest.

The visceral adiposity index (VAI) was defined based on the suggested formula:

Males: VAI = (WC (cm) / [39.68 + (1.88 × BMI (kg/m^2^))]) × (Triglycerides (TG) (mmol/L) / 1.03) × (1.31 / HDL (mmol/L)).

Females: VAI = (WC (cm) / [36.58 + (1.89 × BMI (kg/m^2^))]) × (TG (mmol/L) / 0.81) × (1.52 / HDL (mmol/L)) [39].

Moreover, the waist circumference-triglyceride index (WTI) was calculated as below:

WTI = WC (cm) × TG (mmol/L) [40].

After a 12-hour overnight fasting, venous blood samples were collected at baseline and week 12, and centrifuged at 3000 rpm for 10 min. Then, the sera were stored at -80° C until the final analyses. Commercial enzyme-linked immunosorbent assay (ELISA) kits were used for the quantitative determination of serum endocan and adropin (Crystal Day Biotech Co., LTD, Shanghai, China) and homocysteine levels (Axis-shield, Dundee, United Kingdom). Serum levels of lipid profiles including total cholesterol (TC), triglyceride, high-density lipoprotein cholesterol (HDL-C), and low-density lipoprotein cholesterol (LDL-C) were measured through enzymatic colorimetric methods using commercial diagnostic kits (Pars Azmoon Inc. kit, Tehran, Iran). The 19-item Seattle angina questionnaire (SAQ) was used to assess the clinical status of the patients at baseline and following the intervention. SAQ is a self-administered, valid, reliable, and disease-specific tool sensitive to clinical changes to assess angina status in patients with coronary artery diseases [41, 42]. The questionnaire has 5 domains including physical limitation (9 items), angina stability (1 item), angina frequency and severity (2 items), treatment satisfaction (4 items), disease perception, and quality of life (3 items). Each item has a 5 or 6 numeric scale ranging from 1 to 5 or 6. Scores for each domain were calculated according to the method previously described by Spertus et al. [42]. The scores for each domain ranged from 0 to 100, with higher scores demonstrating better health status.

The average dietary intakes were assessed using a three-day 24-hour dietary recall at baseline and end of week 12. The dietary reported intakes of energy and macronutrients were analyzed using the Nutritionist IV Software (N Squared Computing, San Bruno, CA, USA).

### Statistical analysis

Based on a relevant formula for randomized clinical trials (RCT), the sample size was estimated at 25 patients in each study group, while having 80% power to detect a difference of 5 µmol/L in serum homocysteine levels, with a type I error of 0.05 and drop-out rate of 20% [43]. SPSS software version 26.0 (SPSS Inc., Chicago, IL, USA) was used to perform statistical analyses. The Shapiro-Wilk test was used to explore the distribution of the variables. The data were reported as mean ± standard deviation or frequency (%). To assess statistical differences of baseline values or pre- to post-changes between the intervention and placebo groups, the independent samples t-test for normal continuous, Mann-Whitney test for non-normal continuous or ordinal parameters and Chi-square, and Fisher’s exact tests for nominal parameters were used. The comparison of the outcomes between the two study groups at the end of the trial was performed using analysis of covariance (ANCOVA) adjusted for baseline values. Within-group changes throughout the study were conducted with the Wilcoxon Signed rank test for non-parametric and paired t-test for normally distributed parameters. Statistical significance was defined as a *P*-value < 0.05.

## Results

### General characteristics

**Fig. 1:**
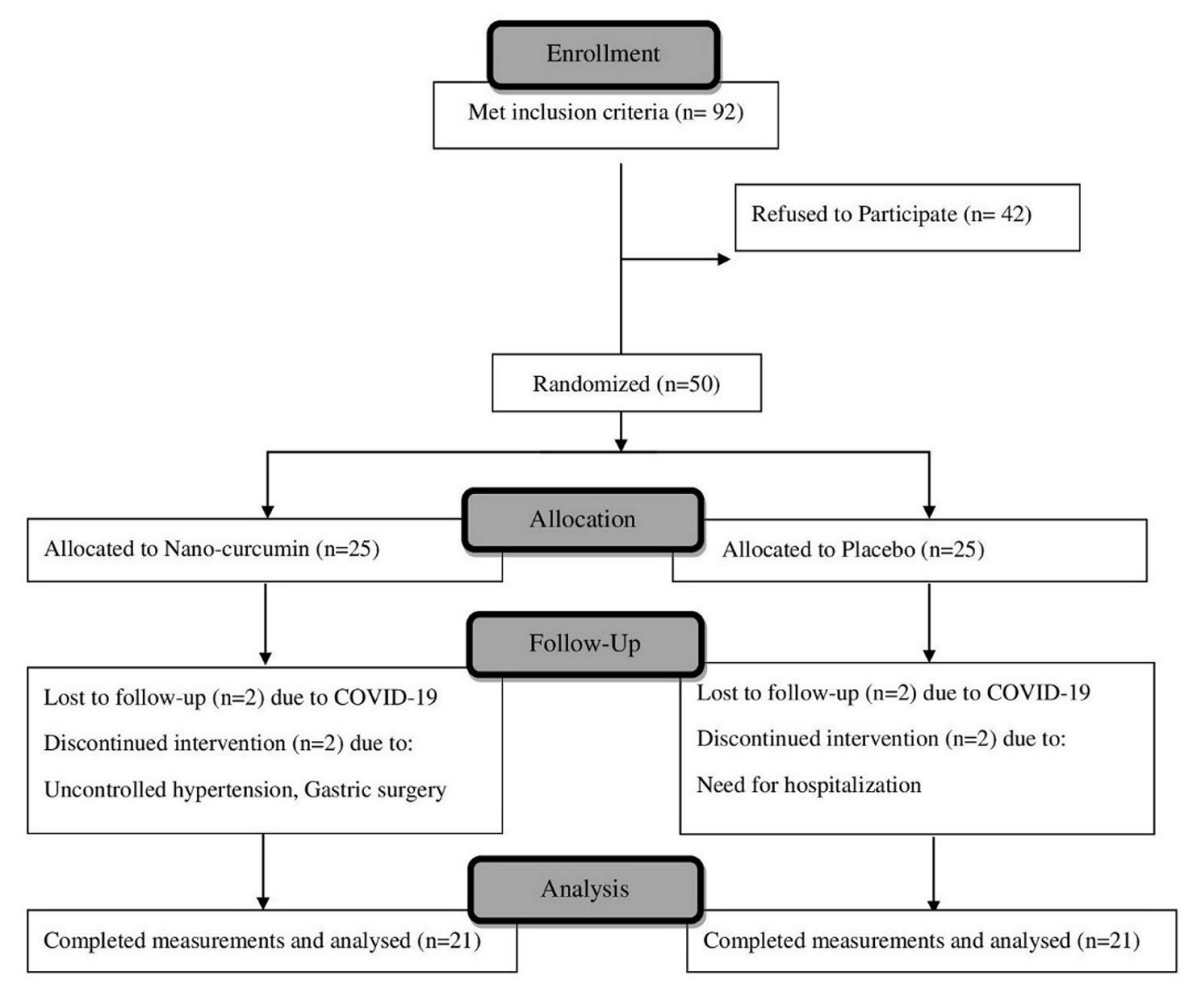
Participant progress during the study of nano-curcumin in CSFP.

Among 92 participants who met the inclusion criteria, 50 patients (25 patients in each group) accepted to participate in this trial and 21 in each group have completed the study. In the nano-curcumin group, two patients were lost to follow-up due to the COVID-19 outbreak, one was excluded due to uncontrolled hypertension, and one due to gastric surgery. In the placebo group, two patients were excluded due to the COVID-19 outbreak and two due to the need for hospitalization (Fig. 1). There were no reports of side effects or intolerance to supplements throughout the study period.

There were no statistically significant differences in age, gender, rate of concomitant diseases such as diabetes, hypertension, and dyslipidemia, and level of blood pressure measurements between the two study groups at baseline (Table 1). Taking medications including aspirin, ACE-I, ARBs, beta-blockers, statins, anticoagulants, calcium channel blockers, and nitrates did not differ significantly between the nano-curcumin and placebo groups (data not shown, all *P* values > 0.05; An additional file is available showing data on medication use in more detail in a supplementary table [see Supplementary Table 1]).

**Table 1:** Baseline characteristics of study participants

		Group		*P*
Parameters		**Placebo** (*n* = 21)	**Nano-curcumin** (*n* = 21)	
**Age (year)**		54.6 ± 8.4	54.3 ± 9.1	0.930 ^a^
**Gender**	Male	17 (81.0%)	17 (81.0%)	1.00 ^b^
**Weight (kg)**		86.3 ± 10.8	85.9 ± 15.3	0.933 ^a^
**BMI (kg/m** ^**2**^ **)**		30.6 ± 3.8	29.7 ± 3.1	0.450 ^a^
**WC (cm)**		102.9 ± 8.3	102.4 ± 11.7	0.850 ^a^
**Percent body fat (%)**		34.5 ± 7.2	33.3 ± 7.1	0.573 ^a^
**SBP** ^¶^ **(mmHg)**		121.2 ± 15.2	121.6 ± 14.2	0.934 ^a^
**DBP** ^**±**^ **(mmHg)**		81.8 ± 10.1	81.8 ± 10.5	0.988 ^a^
**Diabetes**	Yes	6 (28.6%)	8 (38.1%)	0.513 ^c^
**Hypertension**	Yes	10 (47.6%)	8 (38.1%)	0.533 ^c^
**Dyslipidemia**	Yes	15 (71.4%)	12 (57.1%)	0.334 ^c^
**Smoking**	Yes	6 (28.6%)	7 (33.3%)	0.739 ^c^
**Family History of CAD** ^‡^	Yes	10 (47.6%)	15 (71.4%)	0.116 ^c^
**LVEF** ^§^ **(%)**		51 ± 3	51 ± 3	0.941 ^d^
**Number of Slow Flow arteries**				0.239 ^d^
	1 Vessel	9 (42.9%)	5 (23.8%)	
	2 Vessel	7 (33.3%)	9 (42.9%)	
	3 Vessel	5 (23.8%)	7 (33.3%)	

Note: Data are presented as mean ± SD^†^ or frequency (%)

^a^ Independent t-test, ^b^ Fisher’s exact test, ^c^ Chi-square test, ^d^ Mann–Whitney test

^¶^: systolic blood pressure; ^±^: diastolic blood pressure; ^‡^: coronary artery disease; ^§^: left ventricular ejection fraction; ^†^: standard deviation

### Endothelial and cardiovascular parameters

The study parameters investigated in the current trial are presented in Table 2. There were not any statistically significant differences in systolic blood pressure (124.8 ± 12.2 vs. 125.3 ± 11.8 mmHg, *P* = 0.852) and diastolic blood pressure (84.6 ± 7.6 vs. 85.0 ± 12.1 mmHg, *P* = 0.859) between the two groups following the intervention. There were no significant differences in circulating endocan, adropin, homocysteine, and lipid profiles as well as novel cardiovascular risk markers of VAI and WTI between the two study groups at baseline. The differences between groups in serum endocan, adropin, and homocysteine levels remained insignificant after the intervention. Although there were no significant differences between groups in lipid profile at the end of the trial, serum TG levels (186.4 ± 113.8 to 163.6 ± 94.9 mg/dl, *P* = 0.042) and TG/HDL ratio (4.6 ± 3.8 to 3.9 ± 3.1, *P* = 0.039) decreased significantly in the nano-curcumin, but not the placebo group compared to the baseline values. Consequently, significant reductions were also observed in WTI and VAI compared to the baseline values only in the nano-curcumin group (214.0 ± 126.3 to 187.7 ± 104.3, *P* = 0.033 and 2.9 ± 2.2 to 2.4 ± 1.8, *P* = 0.035, respectively).

**Table 2:** Endothelial and cardiovascular parameters of study participants before and after 12 weeks intervention

Parameters	Group		MD ^†^	95% CI ^‡^		*P*
	Placebo (*n* = 21) (mean ± SD)	Nano-curcumin (*n* = 21) (mean ± SD)		Lower	Upper	
**Adropin (ng/l)**						
Baseline	367.3 ± 376.3	309.2 ± 283.3	58.1	-149.7	265.8	0.880 ^a^
Week 12	382.0 ± 411.7	301.9 ± 211.1	80.1	-123.9	284.1	0.369 ^b^
Change	14.7 ± 92.0	-7.3 ± 88.2	22.0	-34.2	78.2	0.910 ^a^
P-within ^c^	0.723	0.175				
**Endocan (ng/l)**						
Baseline	269.0 ± 409.3	264.9 ± 363.4	4.1	-237.3	245.5	1.00 ^a^
Week 12	255.7 ± 404.7	256.1 ± 357.2	-0.4	-238.4	237.7	0.551 ^b^
Change	-13.3 ± 25.6	-8.8 ± 22.8	-4.5	-19.6	10.7	0.428 ^a^
P-within ^c^	0.040	0.260				
**Homocysteine (mcmol/l)**						
Baseline	126.9 ± 234.8	188.0 ± 290.1	-61.1	-225.7	103.5	0.521 ^a^
Week 12	105.6 ± 289.4	211.4 ± 335.0	-105.8	-301.0	89.5	0.431 ^b^
Change	-21.3 ± 269.0	23.4 ± 88.1	-44.6	-169.5	80.2	0.232 ^a^
P-within ^c^	0.614	0.411				
**TC (mg/dl)**						
Baseline	184.6 ± 37.6	179.2 ± 42.7	5.3	-19.8	30.4	0.670 ^d^
Week 12	183.8 ± 40.1	179.8 ± 44.3	4.0	-22.4	30.3	0.922 ^b^
Change	-0.8 ± 26.1	0.6 ± 21.4	-1.4	-16.3	13.5	0.852 ^d^
P-within ^e^	0.888	0.904				
**LDL-C (mg/dl)**						
Baseline	95.3 ± 21.9	87.9 ± 25.6	7.4	-7.4	22.3	0.318 ^d^
Week 12	94.2 ± 22.4	89.9 ± 26.9	4.3	-11.1	19.8	0.660 ^b^
Change	-1.0 ± 15.0	2.0 ± 14.2	-3.1	-12.2	6.0	0.498 ^d^
P-within ^e^	0.753	0.518				
**HDL-C (mg/dl)**						
Baseline	50.9 ± 10.9	49.5 ± 16.2	1.4	-7.2	10	0.571 ^a^
Week 12	50.2 ± 11.4	50.6 ± 15.4	-0.3	-8.8	8.1	0.303 ^b^
Change	-0.6 ± 4.6	1.1 ± 5.4	-1.7	-4.9	1.4	0.307 ^a^
P-within ^c^	0.574	0.314				
**TG (mg/dl)**						
Baseline	157.7 ± 60.8	186.4 ± 113.8	-28.7	-86.1	28.7	0.466 ^a^
Week 12	151.2 ± 69.9	163.6 ± 94.9	-12.4	-64.4	39.6	0.495 ^b^
Change	-6.4 ± 56.3	-22.8 ± 41.4	16.3	-14.5	47.1	0.529 ^a^
P-within ^c^	0.247	0.042				
**TG / HDL**						
Baseline	3.3 ± 1.7	4.6 ± 3.8	-1.3	-3.2	0.6	0.458 ^a^
Week 12	3.2 ± 1.7	3.9 ± 3.1	-0.6	-2.2	0.9	0.397 ^b^
Change	-0.1 ± 1.3	-0.8 ± 1.5	0.7	-0.2	1.6	0.297 ^a^
P-within ^c^	0.339	0.039				
**LDL / HDL**						
Baseline	1.9 ± 0.6	1.9 ± 0.7	0.0	-0.4	0.4	0.898 ^d^
Week 12	1.9 ± 0.5	1.9 ± 0.8	0.0	-0.4	0.4	0.992 ^b^
Change	0.0 ± 0.3	0.0 ± 0.2	0.0	-0.2	0.2	0.993 ^d^
P-within ^e^	0.967	0.973				
**WTI**						
Baseline	186.1 ± 83.3	214.0 ± 126.3	-27.9	-94.6	38.9	0.473 ^a^
Week 12	176.3 ± 83.8	187.7 ± 104.3	-11.4	-70.5	47.6	0.559 ^b^
Change	-9.9 ± 65.6	-26.3 ± 47.6	16.4	-19.3	52.2	0.538 ^a^
P-within ^c^	0.204	0.033				
**VAI**						
Baseline	2.1 ± 1.2	2.9 ± 2.2	-0.7	-1.9	0.4	0.414 ^a^
Week 12	2.1 ± 1.2	2.4 ± 1.8	-0.3	-1.3	0.6	0.326 ^b^
Change	-0.1 ± 0.8	-0.5 ± 0.9	0.4	-0.1	1.0	0.285 ^a^
P-within ^c^	0.455	0.035				

Note: ^a^ Mann–Whitney test, ^b^ ANCOVA, ^c^ Wilcoxon test, ^d^ Independent t-test, ^e^ Paired t-test

ANCOVA: analysis of covariance

^†^: mean difference; ^‡^: confidence interval

### Angina status

There were no significant differences between the study groups in SAQ domains at baseline (Table 3). Following 12 weeks of supplementation, pre- to post-changes of all domains of SAQ including physical limitation (4.5 ± 7.6 vs. -0.7 ± 2.3, *P* = 0.007), angina stability (19.0 ± 24.9 vs. 4.8 ± 10.1, *P* = 0.032), angina frequency and severity (9.5 ± 12.8 vs. 1.0 ± 7.7, *P* = 0.006), treatment satisfaction (11.6 ± 16.0 vs. 1.8 ± 6.9, *P* = 0.004), disease perception and quality of life (6.3 ± 8.7 vs. -0.8 ± 12.6, *P* = 0.039) improved significantly in the nano-curcumin group compared with the placebo group. All subscales of the SAQ were also significantly improved in the nano-curcumin group compared to the baseline status (*P* < 0.05). In the placebo group, only the angina stability score improved compared to the baseline (*P* = 0.046).

**Table 3:** SAQ domain scores of the study participants before and after 12 weeks intervention

Parameters	Group		MD	95% CI		*P*
	Placebo (*n* = 21) (mean ± SD)	Nano-curcumin (*n* = 21) (mean ± SD)		Lower	Upper	
**Physical Limitation**						
Baseline	77.4 ± 13.3	70.8 ± 24.0	6.6	-5.6	18.8	0.278^a^
Week 12	76.7 ± 13.6	75.3 ± 20.3	1.5	-9.3	12.2	0.010^b^
Change	-0.7 ± 2.3	4.5 ± 7.6	-5.2	-8.8	-1.6	0.007^a^
P-within ^c^	0.204	0.014				
**Angina Stability**						
Baseline	66.7 ± 22.8	51.2 ± 36.6	15.5	-3.7	34.6	0.194 ^d^
Week 12	71.4 ± 22.8	70.2 ± 25.8	1.2	-14.0	16.4	0.091 ^b^
Change	4.8 ± 10.1	19.0 ± 24.9	-14.3	-26.3	-2.3	0.032 ^d^
P-within ^e^	0.046	0.004				
**Angina Severity & Frequency**						
Baseline	84.8 ± 11.7	78.1 ± 20.6	6.7	-3.9	17.2	0.416 ^d^
Week 12	85.7 ± 12.5	87.6 ± 13.7	-1.9	-10.1	6.3	0.030 ^b^
Change	1.0 ± 7.7	9.5 ± 12.8	-8.6	-15.2	-2.0	0.006 ^d^
P-within ^e^	0.516	0.003				
**Treatment Satisfaction**						
Baseline	75.6 ± 20.3	67.0 ± 29.1	8.6	-7.1	24.4	0.469 ^d^
Week 12	77.4 ± 18.4	78.6 ± 21.5	-1.2	-13.7	11.3	0.024 ^b^
Change	1.8 ± 6.9	11.6 ± 16.0	-9.8	-17.6	-2.0	0.004 ^d^
P-within ^e^	0.272	0.001				
**Disease Perception & Quality of Life**						
Baseline	60.7 ± 20.6	54.8 ± 32.6	6.0	-11.1	23.0	0.484 ^a^
Week 12	59.9 ± 23.2	61.1 ± 29.4	-1.2	-17.7	15.3	0.054 ^b^
Change	-0.8 ± 12.6	6.3 ± 8.7	-7.1	-13.9	-0.4	0.039 ^a^
P-within ^c^	0.776	0.003				

Note: ^a^ Independent t-test, ^b^ ANCOVA, ^c^ Paired t-test, ^d^ Mann–Whitney test, ^e^ Wilcoxon test

### Dietary intakes and anthropometric indexes

The dietary assessment showed no significant differences in energy intakes, macronutrients, and fiber intakes between the nano-curcumin and placebo groups neither at baseline nor at the end of the trial (Table 4).

**Table 4:** Dietary reported intakes of the study participants at baseline and after 12 weeks intervention

Parameters	Group		MD	95% CI		*P*
	Placebo (mean ± SD)	Nano-curcumin (mean ± SD)		Lower	Upper	
**Energy (kcal)**						
Baseline	2295.3 ± 567.0	2123.1 ± 537.4	172.2	-172.3	516.8	0.318 ^a^
Week 12	2285.1 ± 686.4	2152.2 ± 525.5	132.9	-248.4	514.2	0.617 ^b^
Change	-10.2 ± 308.9	29.1 ± 182.2	-39.3	-197.5	118.9	0.618 ^a^
P-within ^c^	0.881	0.472				
**CHO (g)**						
Baseline	323.1 ± 86.5	285.4 ± 76.6	37.8	-13.2	88.7	0.142 ^a^
Week 12	319.2 ± 105.7	281.1 ± 76.5	38.1	-19.5	95.6	0.942 ^b^
Change	-4.0 ± 53.8	-4.3 ± 36.8	0.3	-28.4	29.0	0.983 ^a^
P-within ^c^	0.738	0.599				
**Protein (g)**						
Baseline	88.1 ± 24.0	87.3 ± 21.2	0.9	-13.3	15.0	0.903 ^a^
Week 12	95.7 ± 27.8	95.6 ± 22.4	0.1	-15.7	15.8	0.860 ^b^
Change	7.6 ± 13.9	8.4 ± 12.7	-0.8	-9.1	7.5	0.850 ^a^
P-within ^c^	0.021	0.007				
**Fat (g)**						
Baseline	73.6 ± 21.9	71.7 ± 23.4	1.8	-12.3	16	0.870 ^d^
Week 12	70.4 ± 22.5	73.1 ± 20.6	-2.7	-16.1	10.7	0.170 ^b^
Change	-3.2 ± 11.3	1.3 ± 9.2	-4.5	-11	1.9	0.232 ^d^
P-within ^e^	0.244	0.664				
**Fiber (g)**						
Baseline	14.6 ± 5.0	14.8 ± 5.3	-0.2	-3.4	3.0	0.910 ^a^
Week 12	15.0 ± 4.3	15.1 ± 5.0	0.0	-3.0	2.9	0.916 ^b^
Change	0.4 ± 3.1	0.3 ± 3.3	0.1	-1.9	2.2	0.884 ^a^
P-within ^c^	0.546	0.711				

Note: ^a^ Independent t-test, ^b^ ANCOVA, ^c^ Paired t-test, ^d^ Mann–Whitney test, ^e^ Wilcoxon test

Anthropometric indexes were not significantly different between the study groups at baseline (Table 1). Additionally, no significant differences were observed in body weight (85.6 ± 15.7 vs. 85.5 ± 9.8 kg, *P* = 0.441), BMI (29.6 ± 3.3 vs. 30.3 ± 3.6 kg/m^2^, *P* = 0.547), WC (102.0 ± 11.5 vs. 102.4 ± 7.9 cm, *P* = 0.788), percent body fat (32.8 ± 7.7 vs. 34.6 ± 6.8%, *P* = 0.239) between the nano-curcumin and the placebo groups at the end of the trial.

## Discussion

The current study investigated the effect of a nano-curcumin supplement which contained curcuminoids on novel endothelial biomarkers potentially implicated in CSFP pathogenesis, cardiovascular risk factors, and angina status as a measurement of clinical endpoint among overweight or obese patients with CSFP. The results showed that 80 mg/day nano-curcumin supplementation for 12 weeks can significantly improve the clinical status of people with CSFP compared to the placebo, without affecting the main assumed contributing factors such as homocysteine, endocan and adropin levels.

To our knowledge, changes in the clinical aspects of CSFP have not been previously investigated following nutritional supplementations. At the end of the current trial, the frequency and severity of anginal episodes and treatment satisfaction were significantly better in the nano-curcumin than the placebo group. Nano-curcumin has also significantly improved physical limitations due to angina, angina stability, disease perception, and quality of life in the intervention patients compared with the placebo group. Curcumin supplementation has shown promising effects on different mental and physical aspects of quality of life such as bodily pain, disease severity, fatigue, well-being, vitality, and depression in various medical conditions including mild hypertension, obesity, liver cirrhosis, and irritable bowel syndrome [30, 31, 44, 45]. Part of the beneficial effects of curcumin on better clinical outcomes and general health, based on SAQ, might be attributed to reducing inflammation through significant improvement in inflammatory markers [44]. Additionally, it has been shown that psychological disorders could largely induce different physical symptoms and problems [46]. A recent meta-analysis study showed that curcumin could improve depressive and anxiety symptoms [47]. Thus, probably psychological improvements following nano-curcumin supplementation had a role in improving the disease-associated physical and mental status and SAQ scores.

Curcumin has pleiotropic activities; besides targeting pro-inflammatory cytokines, it can modulate adhesion molecules, antioxidant enzymes, and endothelial mediators which all are directly involved in endothelial function [18, 26, 48, 49]. Apart from curcumin, other minor curcuminoids also have been shown to possess various biological activities in several in-vitro and in-vivo studies; and in some cases, demethoxycurcumin and bisdemethoxycurcumin have shown more potent effects than curcumin [20, 50]. In a recent in-silico and in-vitro study, curcuminoids including curcumin, demethoxycurcumin and bisdemethoxycurcumin have shown to inhibit the tubulogenic and migration capacity of endothelial cells and reduced phosphorylation of the VEGFR2 in VEGF-165-stimulated cells suggesting an anti-angiogenic property [50]. Moreover, bisdemethoxycurcumin has shown a novel anti-inflammatory pathway by inducing expression of heme oxygenase-1 and attenuating inducible NOS expression [51]. The supplement used in the current study contained several curcuminoids.

However, in the current study, the nano-curcumin supplementation did not lead to any significant improvements in serum endocan levels, as a possible mediator of endothelial dysfunction in comparison to the placebo. No previous study was available investigating the effect of nano-curcumin or other similar nutraceuticals on serum endocan in patients with CSFP. Few clinical studies are currently available describing the behavior of endocan and the mechanisms of its regulation in health and diseases. Some data suggest that endocan may play a role in the development of CSF [15], while others propose that endocan may be an endpoint, rather than a cause, of endothelial inflammation, activation, and dysfunction [52]. Accordingly, effective targeting of this mediator and the consequent clinical benefits need further investigations.

Adropin is a novel regulator of endothelial function, which can be expressed by endothelial cells [12]. Previous observational studies have shown decreased levels of serum adropin in patients with CSFP and its potential predictive value for the presence of this condition [7]. In the present study, nano-curcumin supplementation did not change adropin levels significantly neither between nor within the two groups. There is no comparable study available in CSFP or other cardiovascular diseases. Adropin level is inversely associated with body weight, and insulin resistance [53]. Previous clinical trials showed a concomitant decrease in body fat and insulin resistance and an increase in serum adropin following low-calorie diet supplemented with probiotic yogurt or aerobic exercise [54, 55]. However, in the present trial, there was a heterogeneity in terms of insulin resistance among the participants. Additionally, despite being overweight or obese, body weight and fat percent did not change significantly following nano-curcumin supplementation in the study participants. These discrepancies along with differences in the pathophysiological background of the previous studies might partly explain the lack of significant changes in circulating adropin in the current study.

Elevated levels of homocysteine have been reported in CSFP patients, which is positively correlated with mean TFC and negatively with FMD [8, 56]. Increased homocysteine levels cause direct damage to the endothelium and reduce NO bioavailability, increase TG levels, LDL-C oxidation and reduce HDL-C [57, 58] In the current study, nano-curcumin supplementation did not significantly change homocysteine levels compared with the placebo. No similar study was available in CSFP. However, Latif et al., also found no significant changes in serum homocysteine levels in overweight or obese women after 2 g/day turmeric supplementation for 90 days [59]. In contrast, 12 weeks of supplementation with 500 mg/day of a highly bioavailable formulation of curcumin in healthy people with BMI ≥ 30 kg/m^2^ led to significant reductions in homocysteine levels compared with the placebo group [60]. Homocysteine has a complicated metabolism and several factors influence its concentrations [57]. Discrepancies observed in homocysteine response to curcumin supplements in different studies may, at least in part, be related to differences in underlying medical conditions, concomitant metabolic disorders, as well as doses, absorption rate, bioavailability and bioactive constituents of the curcumin supplements.

TG/HDL-C ratio is correlated with cardiovascular events and is a feasible index of atherogenic dyslipidemia in clinical practice [61]. Recently it has been reported that TG/HDL ratio is significantly elevated in patients with CSFP and it is an independent predictor of the presence and severity of the disease [28]. A trial in diabetic patients showed no significant changes in lipid profile, while a significant improvement was observed in HDL-C, TC, TG, and LDL-C in patients with non-alcoholic fatty liver disease compared to the control group following nano-curcumin supplementation [33, 34]. These differences in the results might be partly due to the higher efficacy of nano-curcumin, like many other treatments, to change cardiometabolic risk factors when the baseline values are impaired. Moreover, Rastmanesh et al. recently showed that the intra-individual differences in terms of dietary intake and food processing techniques result in differential bioactivity and bioavailability of many antioxidants including curcumin and curcuminoids [62]. They also added that even randomization protocols or using cross-over or parallel designs cannot eliminate this source of bias [62].To our knowledge, dietary intakes of curcumin and curcuminoids have not been assessed in previous studies as well as the current trial. Serum concentrations of these compounds have not been evaluated, either; while the effects of these compounds are greatly influenced by their concentrations. This might affect the results and partly explain the differences in findings of different studies.

Obesity is one of the major conventional risk factors for various cardiovascular diseases including CSFP [63]. Recently, increasing interest has been directed toward developing combined scores consisting of both indicators of obesity and biomarkers, to better reflect the clinical status [39]. VAI is an indicator of fat distribution in the visceral or subcutaneous area and it is of high predictive power for cardiovascular risk [39]. An increase in VAI shows adipose tissue dysfunction and a reduction in insulin sensitivity [39]. WTI has been also suggested for predicting coronary artery disease [40], as it has a direct correlation with the chance of coronary artery lesions and coronary heart disease [64]. In the present study, nano-curcumin supplementation reduced VAI and WTI significantly compared with the baseline values, which was simultaneous with better clinical scores of SAQ in the intervention group. Thus, it seems these novel indexes may show better overall cardiovascular health and less adipose tissue dysfunction in CSFP patients and are worth to be explored in future studies in this field.

This study had some limitations, which should be noted. Due to ethical concerns, it was not possible to repeat angiography, as the gold standard tool for investigating clinical and functional changes of coronary arteries, at the end of the 12-week nano-curcumin supplementation. Additionally, investigating FMD, as the most popular non-invasive method for the assessment of endothelial dysfunction, would better clarify the lack of significant changes in the biomarkers following the intervention. In addition, we did not investigate the potential long-term effects of supplementation of nano-curcumin on our primary and secondary outcomes. Therefore, further trials with longer durations are needed to test the long-term effects of supplementation with nano-curcumin. Moreover, in spite of asking participants to follow their routine diet, the baseline dietary intakes of curcumin and curcuminoids and their intakes throughout the study were not quantitatively assessed due to the lack of a valid semi-quantitative questionnaire to assess the intake of these bioactive compounds. Conducting further well-designed trials with larger sample sizes and longer trial durations while exploiting valid questionnaires to assess dietary intakes of curcumin and curcuminoids should be also considered to increase the power of the study in detecting the potential efficacy of nano-curcumin in CSFP.

## Conclusions

The results of the current trial showed that 12 weeks of supplementation with 80 mg/day nano-curcumin supplement (containing curcuminids including curcumin, demethoxycurcumin, and bisdemethoxycurcumin) could improve disease-related physical and mental complications including angina stability, frequency and severity, physical limitation as well as treatment satisfaction and disease perception and quality of life, in overweight or obese patients with CSFP compared with the placebo group. Additionally, TG, TG/HDL ratio, and novel indexes WTI and VAI, improved significantly within the nano-curcumin, but not the placebo group, compared to the baseline values. No significant changes were observed in serum endocan, adropin, and homocysteine following the supplementation. Overall, oral nano-curcumin supplementation seems to be a simple, well-tolerated complementary treatment for improving general health and anginal episodes. Further studies are needed to explore the possible mechanisms involved in both the pathogenesis of CSFP and the beneficial effects of nutraceuticals such as curcumin.

### Electronic supplementary material

Below is the link to the electronic supplementary material.

Additional file 1 Table 1. Drug history of the participants at the beginning of the study.

## Data Availability

The data that support the findings of this study are available on request from the corresponding author. The data are not publicly available due to privacy or ethical restrictions.

## Abbreviations

CSFP: Coronary slow flow phenomenon
SAQ: Seattle angina questionnaire
ANCOVA: Analysis of covariance
WTI: Waist-triglyceride index
VAI: Visceral adiposity index
NO: Nitric oxide
NOS: Nitric oxide synthase
CTFC: Corrected thrombolysis in myocardial infarction frame count
BMI: Body mass index
WC: Waist circumference
ACE-Is: Angiotensin-converting enzyme inhibitors
ARBs: Angiotensin II receptor blockers
FMD: Flow mediated dilation
TG: Triglyceride
LDL-C: Low-density lipoprotein cholesterol
HDL-C: High-density lipoprotein cholesterol
TC: Total cholesterol
