# Supplementary material for: Effect of nano-curcumin supplementation on angina status, and traditional and novel cardiovascular risk factors in overweight or obese patients with coronary slow flow phenomenon: a randomized double-blind placebo-controlled clinical trial
Source: BMC Nutr. 2024 May 13;10:73. doi: 10.1186/s40795-024-00877-3 (PMC11089698; doi:10.1186/s40795-024-00877-3)
Supplement: Supplementary file 1 — Additional file 1 Table 1. Drug history of the participants at the beginning of the study. [file 40795_2024_877_MOESM1_ESM.docx]

| **Supplementary Table 1**. Drug history of the participants at the beginning of the study. | | | | |
| --- | --- | --- | --- | --- |
|  | | **Group** | | **P** |
| **Parameters** | | **Placebo** (n = 21) | **Nano-curcumin** (n = 21) |  |
| **Nitrates** | Yes | 0 (0.0%) | 3 (14.3%) | 0.23 ^a^ |
| **ACE-Is** ^¶^ | Yes | 3 (14.3%) | 1 (4.8%) | 0.61 ^a^ |
| **Aspirin** | Yes | 14 (66.7%) | 17 (81.0%) | 0.29 ^b^ |
| **ARBs** ^±^ | Yes | 5 (23.8%) | 7 (33.3%) | 0.50 ^b^ |
| **Statins** | Yes | 15 (71.4%) | 13 (61.9%) | 0.51 ^b^ |
| **CCBs** ^‡^ | Yes | 4 (19.0%) | 4 (19.0%) | 1.00 ^a^ |
| **BBs** ^§^ | Yes | 6 (28.6%) | 5 (23.8%) | 0.73 ^b^ |
| **Anticoagulants** | Yes | 1 (4.8%) | 2 (9.5%) | 1.00 ^a^ |
| Note: Data are presented as frequency (%)  ^a^ Fisher's exact test, ^b^ Chi-square test  ^¶^: Angiotensin-Converting Enzyme Inhibitors; ^±^: Angiotensin II Receptor Blockers; ^‡^: Calcium Channel Blockers; ^§^: Beta Blockers | | | | |

**Supplementary materials**
